# Letter from China

**Published:** 1881-05

**Authors:** Kate C. Bushnell

**Affiliations:** Kiu Kiang, China


### Foreign Correspondence.

### ARTICLE IX.

KIU KIANG, CHINA, Feb'y 25, 1881.

MESSRS. EDITORS:—Previous to my departure for China, I gave you a promise to write to your JOURNAL in regard to the *native medical practice* of China. I have delayed so long on account of the impossibility of gaining any definite idea of the subject until I had acquired a knowledge of the language of this country, and even now I hesitate to impart the little knowledge I have gained, for fear it may not have come from the most reliable sources.

I have in my circle of native friends three literary gentlemen who practice medicine. One of them is a member of a Christian church, and seems to have great confidence in foreign practitioners, and occasionally comes to me with patients whom he is unable to cure.

From these native physicians I have learned many things in regard to their methods of treatment and knowledge of general medicine, and I will now relate a few of these facts, giving you the privilege of printing any or all of them, as you see fit.

The native physician of China is a member of the literary class, and well versed in the philosophy of Confucius—of medicine he is not required to have any special knowledge. There is a law by which physicians and druggists are held responsible for errors in prescriptions, but the native never takes advantage of it.

The creation of a physician generally comes about in the following manner: A Chinaman is seriously ill; he sends for the literary relative (or relatives, as the case may be) of the family for advice. The literary gentleman searches all the works on medicine at his disposal, and treats the patient according to directions. Should that literary man happen to be successful in his treatment, his fame is spread abroad, and ever after that he is consulted as a specialist in that particular disease. There is no doubt that many a man of the most unreliable character makes his way to the front of the medical ranks in this way, and doubtless many a life is sacrificed thereby.

The *last resort*, in serious cases of sickness, is that of administering fresh warm human flesh to a dying person, and it is said there are many monuments in China erected to the memory of women who have allowed themselves to be butchered for their husbands, brothers or fathers. I have yet to learn of a case where either the dying man or the person from whom the live flesh was cut recovered; unfortunately, however, the *physician* usually survives the operation. Fortunately this custom is not a common one.

Consumption, dropsy, obstinate vomiting, and insanity, are considered incurable by native physicians.

Practice is divided into (1) Outside, (2) Inside, (3) Diseases of Women, (4) Diseases of Children, and (5) Eye Diseases.

*Outside* diseases comprise skin diseases, abscesses, burns, fractures, and all other diseases that are apparent on the surface of the body.

*Inside* diseases comprise all those that are not apparent on the surface, excepting those mentioned below, and are diagnosticated by the pulse.

*Diseases of Women* comprehend any kinds of ill the female is afflicted with—be it uterine or otherwise—excepting *outside* diseases.

*Diseases of Children* and *Eye Diseases* indicate the same disorders that we comprehend under these titles.

Anatomy and physiology are comparatively unimportant studies for the native physician. Not long since I asked a physician where the liver was situated, and he was undecided as to whether it was on the right or left side.

The human body is divided into five organs and six viscera. The five organs are the heart, lungs, spleen, liver and kidneys; the six viscera are the stomach, gall-bladder, intestines and three functional passages (rectum, vagina and urethra.)

Very little is said of muscles, tissues, bones and joints. Blood-vessels are supposed to be channels through the flesh. In reply to my question to a physician, "How do you account for motion in a part?" he said, "The blood and the breath go into the part and cause it."

Nothing is known of nerves. The brain is called "a great sea of marrow," and controls the marrow of the bones. Marrow is very essential to health. In childhood it is scant, in healthy middle-age very abundant, and in old age the bones are hollow. If a child cannot walk, it is because the bones of the legs contain no marrow.

The heart is the seat of thought and moral character. Many physicians believe the heart possesses even eyes. These eyes are very brilliant in intelligent people, and very sleepy in stupid ones. It has been gravely decided that the head must be the seat of the memory, because people have been observed to scratch the head when trying to recall events of past time. The heart rules over the blood, the brain over the marrow, the spleen over the flesh, and the kidneys over the bones. The *life* is a small *organ* situated between the kidneys. Chemistry is an unknown science. The elements of the universe are metal, wood, water, fire and earth. The heart is controlled by fire, the liver by wood, the lungs by metal, the spleen by earth and the kidneys by water. The organs of speech are situated in the lungs.

The liver hoards up the blood. The heart and the small intestine are said to be intimately associated. The lungs have a close relation to the large intestine. The kidneys control the bladder, though the natives know of no connection between the two. The spleen governs the stomach and the liver rules over the gall-bladder. The process of digestion is as follows: There are two tubes opening into the pharynx—one from the lungs and another passing to the stomach. The food passes down the latter tube into the stomach, and keeps up a constant motion, by means of which the food is digested. Thence the food passes into the small intestine, when the good food passes into the blood-vessels. The waste matter proceeds to the large intestine, from whence the waste *solid* matter goes to the rectum, and the waste *fluid* matter to the bladder.

In materia medica the varieties of drugs are very extensive, and their application as various as would naturally result from the irregularities of the native physicians. Sulphur is used for scabies, and mercury for syphilis. Arsenic is used as a caustic, though strictly forbidden for skin diseases. Tiger's flesh and bones are used for making tonics. Not many weeks ago a huge beast of this sort was brought to me in the hope that I would purchase it for my dispensary. It had been shot in the mountains not far distant from Kiu Kiang. The fæces of various animals, as well as crystals prepared from urine, are common medicines. A vegetable oil is administered frequently in consumption. Musk is said by the natives to be stimulating, antispasmodic, expectorant and anthelmintic. Sugar-water is the domestic remedy among the natives for vomiting. Vermillion is used as an antispasmodic. I have seen two cases of paralysis this year, due, it was claimed, by the patient's too powerful medicines, administered by a native physician—one case was said to have resulted from the use of vermillion. Unfortunately circumstances did not permit me to make a very thorough examination of either case.

Undoubtedly the most interesting department of native practice is therapeutics—it is also in this line that one might learn a few things of value to the general medical profession, had one time and opportunity to search in this direction. However the value of such research is readily overestimated, unless one considers the utter lack among the natives of real knowledge of the fundamental branches of medicine, without which but little advance can ever be made. The diagnosis of internal diseases by means of the pulse, is reduced to an exact science. The principal directions for conducting examinations are as follows: If the patient be a male, feel the left (the side of honor) pulse first; if it be a female, feel the right pulse first. Supposing one is dealing with a male: place the first, second and third fingers on the radial pulse of the left side, at some distance apart if he be an adult, close together if it be a child.

The first finger distinguishes diseases of the heart and diaphragm ; the middle finger diagnosticates diseases of the liver and gall ; and the third finger diseases of the kidneys, small intestine and bladder. Now, placing the fingers on the right radial pulse, the first finger distinguishes diseases of the lungs and chest ; the middle finger, diseases of the stomach and spleen ; and the third finger, the *life*, large intestine, and general condition of the body.

There are four main divisions of the pulse—a floating, sunken, quick and slow pulse. These constitute four headings, that are subdivided into twenty-seven different varieties, such as large, small, coarse, fine, etc.

Uterine trouble is recognized, but no varieties of the disease are distinguished, so far as I am able to learn. It is treated as a symptom of a general anæmic condition, and tonics administered therefor. There is no doubt that uterine disorders of all kinds are exceedingly common in China. Native physicians say ninety per cent. of the women are troubled with leucorrhœa.

An "ague nest" of phlegm and blood is said to form in the left side of patients suffering from malarial poisoning. It is pro nounced incurable, and is in close connection with the liver.

Tumors of the abdomen are *real* or imaginary. There is a variety of tumor which forms in the abdomen of men who are given to strong drink. Sometimes it comes out of the mouth during life, and it resembles a snake, centipede or mud-turtle !

There is also a large tumor mentioned in native works, which grows on the lower part of the back (*exceedingly rare !*) and when it is cut open, live pediculi escape !

Auscultation and percussion are never practiced. Only the most skillful physician can differentiate ascites and tympanitis. A short time ago a young boy was brought to me with acute Bright's disease. As his life was endangered by an immense accumulation of fluid in the abdomen, and every other means of removing the fluid failed, I performed paracentesis—to the great astonishment of native physicians and the friends of the family. I was afterwards closely questioned in regard to how I knew it was water instead of gas in the bowels, as all the native physicians who had seen the case had been unable to decide.

The natives divide consumption into that of the lungs, heart, liver, spleen and kidneys. In all these forms it is considered incurable.

Not long since a babe, one and one-half years old, was brought to me for treatment after the native physicians had given up the case. They had pronounced it a case of consumption, and fed the babe on stewed pig's liver. I learned that the mother's milk had failed several months before, and the child was being raised (according to Chinese custom) on rice-water. The babe eagerly devoured everything in the shape of food presented to her, and on beef and chicken broth, eggs and cows' milk was restored to good health in two weeks.

Paralysis is said to be due to three causes: (1) a wind suddenly blows upon a part and dries up its juices; (2) the blood no longer flows to the part; (3) there is some obstacle to the free entrance of breath to the part.

Insanity is possession by an evil spirit, the result of mental excess, or the result of an accumulation of the juices of the body between the heart and diaphragm.

Convulsions are due to an accumulation of juices about the heart. No differentiation is made between epileptiform convulsions, apoplexy and syncope.

Generally a person is considered dead when he faints, and when he recovers, he is restored to life again.

There are two varieties of convulsions in children, *slow* and *sudden* (acute and chronic). The latter variety is easily cured; the former variety is due to an inherited dyscrasia, and cannot be cured, but slowly spreads to the spleen, and in spite of caustics, pinching, purging and tonics, the child eventually dies. The method of distinguishing the two varieties is as follows: Either take hold of the hand or foot and shove forward the integument of the palm or sole (as the case may be) with the thumb, or gouge the flesh of the knee, elbow or furrow in the upper lip, with the thumb-nail, or pick up the skin in these places and break the skin connection.

In case these modes of treatment do not cure the child, use the caustic treatment. This consists in twisting a bit of red paper to a point, then dip it in gesamum oil, light it and touch it to the flesh in various parts. If it be a case of sudden fit, the child will quickly recover thereafter; if the child does *not* flinch from the fire the case is incurable.

The regulation of the diet is a very important consideration with the native physician. Melons are considered fatal in effect if eaten in any disease. In diarrhœa and fever, meat and oil are prohibited. In cases of abscesses one must be careful about eating articles of food that *work* like yeast. To this class belong fish, flour, chickens, and more especially pumpkins. In dropsy no salt is allowed for a full year. In consumption *white ducks* are allowed, but no chicken nor fish. For indigestion, eat arrow-root, flour and soft cooked rice. A few lumbricoids in the alimentary canal are considered very helpful for digestion.

Surgery is limited, mostly, to the opening of abscesses and the extraction of teeth. A fracture is never treated so that the part regains its full use. The use of sutures in extensive wounds is unheard of except in the neighborhood of foreign practitioners. Last summer, having an occasion for sewing up a wound, I did so, to the great astonishment of the natives, who speedily spread the report, "She can make seams in the human flesh! Who ever heard of such a thing before?"

I have been assured by the natives that a felon can be aborted by the early application of a pig's gall-sac, and that it quiets the pain almost instantly.

The department of obstetrics is left entirely in the hands of midwives, and consequently many a woman dies for the want of medical aid. During the month of January last, I was called to two cases where the midwives had failed in effecting delivery.

Case No. 1 was readily relieved by aid of forceps, and was doubtless the result of inattention to the bladder, which was enormously distended with urine.

Case 2 was a shoulder presentation. The liquor amnii had escaped two days before I was called, and decomposition had set in, in the protruding arm. In this case embryrotomy was the only resort practicable. The mother made a good recovery.

In order to show the varieties of diseases one meets with in

China, I will add that in the past year I have treated cases as follows:

Diseases of respiratory system, 70; diseases of alimentary system, 83; diseases of nervous system, 8; febrile disorders, 122; urinary and renal, 7; heart diseases, 9; miscellaneous medical, 62; gynæcological and obstetrical, 42; eye and ear, 115; skin, 59; surgical, 76; venereal, 122.

Leprosy is rarely seen in this province of China.

In conclusion, I would like to say, that if you have any charitably disposed Christian medical students who would find it a pleasure to heal the sick and preach the gospel to the poor, they can find no better opportunities for doing both than China, and a medical missionary's life offer. But, alas, I fear many would prefer to stay in America and soar as high as their ability permits, and all this self-denial (of a very extensive practice and opportunities that home can never afford), they make for fear of running athwart that time-honored saying, "Charity begins at home."

If you could suggest any particular lines of investigation that could be carried on without expenditure of any great amount of time, I should be pleased to follow your suggestions, both for my own sake and for the opportunity of contributing something interesting to the columns of your journal.

For lack of time in which to make a carefully written article for the press, I write simply a letter, allowing you to choose therefrom that which is worthy of publication.

Very sincerely yours,

KATE C. BUSHNELL.

---

EPIDEMIC WINTER DIARRHŒA.—This has prevailed in this and other cities East and West lately. It is the general impression with our local physicians that it is a follicular enteritis. As to treatment, the universal testimony is in favor of the free use of bismuth. Anodynes and mild astringents are also useful. No death from that disease is reported.

